# Ethanol Induces Craniofacial Defects in Bmp Mutants Independent of *nkx2.3* by Elevating Cranial Neural Crest Cell Apoptosis

**DOI:** 10.3390/biomedicines13030755

**Published:** 2025-03-20

**Authors:** Hieu D. L. Vo, C. Ben Lovely

**Affiliations:** Department of Biochemistry and Molecular Genetics, University of Louisville School of Medicine, 580 S. Preston St., Louisville, KY 40202, USA; vo.hieu@louisville.edu

**Keywords:** fetal alcohol spectrum disorders, zebrafish, alcohol, genetics, endoderm, craniofacial development

## Abstract

**Background:** Craniofacial malformations lie at the heart of fetal alcohol spectrum disorders (FASDs). While there is growing evidence for a genetic component in FASDs, little is known of the cellular mechanisms underlying these ethanol-sensitive loci in facial development. The bone morphogenetic protein (Bmp) signaling pathway-dependent endoderm pouch formation is a key mechanism in facial development. We have previously shown that multiple Bmp mutants are sensitized to ethanol-induced facial defects. However, ethanol does not directly impact Bmp signaling. This suggests that downstream effectors, like *nkx2.3*, may mediate the impact of ethanol on Bmp mutants. **Methods:** We use an ethanol exposure paradigm with *nkx2.3* knockdown approaches to test if *nkx2.3* loss sensitizes Bmp mutants to ethanol-induced facial defects. We combine morphometric approaches with immunofluorescence and a hybridization chain reaction to examine the cellular mechanisms underlying Bmp–ethanol interactions. **Results:** We show that Bmp–ethanol interactions alter the morphology of the endodermal pouches, independent of *nkx2.3* gene expression. Knockdown of *nkx2.3* does not sensitize wild-type or Bmp mutants to ethanol-induced facial defects. However, we did observe a significant increase in CNCC apoptosis in ethanol-treated Bmp mutants, suggesting an ethanol sensitive, Bmp-dependent signaling pathway driving tissue interactions at the heart of FASDs. **Conclusions:** Collectively, our work builds on the mechanistic understanding of ethanol-sensitive genes and lays the groundwork for complex multi-tissue signaling events that have yet to be explored. Ultimately, our work provides a mechanistic paradigm of ethanol-induced facial defects and connects ethanol exposure with complex tissue signaling events that drive development.

## 1. Introduction

Fetal alcohol spectrum disorders (FASDs) are a major issue in the US, impacting up to 5% of live births, resulting in severe societal impact and making prenatal alcohol exposure (PAE) the leading preventable cause of birth defects [1,2]. FASD is an umbrella term describing a range of possible diagnoses for PAE individuals, amongst which FAS is at the most severe end of the spectrum, encompassing a wide range of adverse effects including defects to the facial skeleton [1]. Hallmark facial features of PAE include a reduced palpebral fissure length, smooth philtrum, thin upper vermillion lip border and, in this manuscript, an underdeveloped jaw [1]. Ethanol dosage and timing and genetic predisposition contribute to FASD etiology [1,2]. Genetics play a key role in FASD etiology, with the best evidence being human twin studies, where there is 100% concordance in FASD phenotypic outcomes in monozygotic twins over dizygotic twins (66%), full siblings (41%), and half siblings (22%) [3]. Animal models have built on this with several ethanol-sensitizing alleles in zebrafish and mice that link with multiple aspects of development, including increased cell death, holoprosencephaly, disruption to axonal projections, and broad neural and eye defects [4,5,6]. In particular, multiple genes have now been identified that sensitize animal models to ethanol-induced craniofacial defects [7]. However, genetic predisposition and, more importantly, the cellular and molecular events they regulate that give rise to facial defects in FASDs are still poorly understood.

Zebrafish is an excellent animal model to study gene–ethanol interactions due to its genetic tractability, external fertilization, and translucent embryos. In addition, external fertilization allows us to precisely control the timing and dosage of ethanol [8]. Zebrafish embryos develop rapidly, with the craniofacial skeleton able to be labeled and visualized 4–5 days post fertilization (dpf). Genetic manipulation with powerful techniques such as CRISPR/Cas mutagenesis and Tol2 transgenesis allows us to readily generate mutant and transgenic lines, respectively. Due to its high fecundity, zebrafish studies can easily generate hundreds of these genetically manipulated embryos enabling high-throughput analysis. This, combined with an easy visualization of the embryos using 4D confocal imaging techniques, demonstrates that zebrafish can quite readily generate a large volume of meaningful data. Moreover, due to genetic conservation, these studies will be directly applicable to humans, since over 70% of genes in zebrafish have homologs in humans, with this percentage increasing to 82% when the genes are known to cause human diseases [8,9]. While being a strong model for humans, zebrafish do possess a handful of limitations. Genetically, zebrafish are tetraploid and highly inbred, lacking the variation observed in human populations, which complicates genetic comparisons with humans. In addition, the structures of the zebrafish facial skeleton are not homologous to humans, lacking several structures common in mammals, for example, a secondary palate. However, the genetic regulation of palate formation in zebrafish is still conserved to mammals [10]. While not perfect, the zebrafish is an ideal model in which to study genetic and environmental factors, as well as their interactions, in craniofacial malformations.

In all vertebrates, the craniofacial skeleton is derived from cranial neural crest cells (CNCCs), which originate from the dorsal neural tube and migrate and condense in the pharyngeal arches [8,11,12]. However, proper facial development can only be achieved by complex interactions between the CNCCs and the surrounding facial epithelia, particularly the pharyngeal endoderm [11]. While the CNCCs are undergoing their migration, cells of the pharyngeal endoderm also undergo their own morphogenesis to form lateral protrusions called pouches [12]. This pouch-forming process involves subsets of endodermal cells reorganizing their points of cell adhesion and following a migratory cue to out-pocket and form the pouches [13,14]. These pouches then segment the pharyngeal arches and provide scaffolding as well as signaling centers that guide the CNCCs throughout facial development [15]. Perturbations to pouch morphogenesis disrupt their signaling interactions with the CNCCs, resulting in defects to the facial skeleton [12,13,14]. Together, the endodermal pouches and their interactions with the CNCCs are indispensable for craniofacial development [12,16].

Multiple genetic signaling pathways have been shown to regulate pouch morphogenesis, including the bone morphogenetic protein (Bmp) signaling pathway [12,14,17,18]. We have previously shown that Bmp signaling is critical for the pouch formation, with disruptions in pouch development leading to severe craniofacial defects [12]. The inhibition of Bmp signaling with the small chemical inhibitor Dorsomorphin (DM) from 10 to 18 hpf reduces fibroblast growth factor (Fgf) signaling responses in the endoderm by regulating the expression Fgf receptor, *fgfr4* [12]. Previous studies have also shown that Bmp signaling is also required for the specification of a pre-pouch endoderm through the expression of Homeobox protein transcription factor NK2 homeobox 3, *nkx2.3*, and that the loss of *nkx2.3* disrupts the formation of the posterior craniofacial cartilages, in part by regulating the expression of the Fgf ligands, *fgf3* and *fgf8a* [17,19]. An examination of the timing of gene expression in both wild-type and Bmp knockdown showed that *nkx2.3* is expressed and impacted by Bmp signaling loss earlier than Fgf signaling, placing *nkx2.3* upstream of Fgf in pouch development [12,17,19]. Overall, this suggests that a complex Bmp-*nkx2.3* signaling pathway regulates pouch development and establishes Bmp signaling as a key regulator of early endoderm development that is critical to subsequent craniofacial development.

As an important pathway in early craniofacial development, Bmp signaling is a strong candidate as a target of ethanol sensitivity driving ethanol-induced craniofacial defects. We have recently shown that multiple Bmp mutants (the ligands *bmp2b* and *bmp4* and the receptor *bmpr1bb*) are sensitive to ethanol-induced facial defects when exposed during the DM-sensitive time window, 10–18 hpf [20]. We show that ethanol-treated *bmp4*; *smad5* (Mothers Against DPP Homolog 5) double mutants exhibit increases in the size of the anterior endoderm, the disruption of *fgf8a* expression in the oral ectoderm, and subsequent severe facial defects, including those in the posterior cartilage elements [20]. Strikingly, we observed that Bmp signaling responses are not sensitive to ethanol [20]. This suggests that Bmp–ethanol interactions disrupt endoderm morphogenesis, possibly via downstream targets like *nkx2.3*. However, the expression of *nkx2.3* in ethanol-treated Bmp mutants and its role in ethanol-induced pouch and facial defects in Bmp mutants remain unanswered.

Here, we investigate the Bmp downstream target, *nkx2.3*, to determine the mechanism of ethanol action on pouch morphogenesis and subsequent craniofacial development. We show that ethanol does not alter the expression of *nkx2.3* in wild-type or *bmp4*; *smad5* double-mutant embryos. We go on to show that *nkx2.3* morphants are not ethanol-sensitive. In addition, *nkx2.3* knockdown does not further sensitize *bmp4* single-mutant embryos to ethanol-induced craniofacial defects. This suggests that ethanol is acting entirely independent of Bmp signaling and its downstream target, *nkx2.3*. Previous work has shown that the CNCCs are sensitive to ethanol-induced apoptosis [21], suggesting that increased CNCC apoptosis may be driving the facial defects in our ethanol-induced Bmp mutants. Here, we show that our Bmp–ethanol interaction significantly increases CNCC apoptosis in ethanol-treated Bmp mutants. Overall, this suggests a two-hit model, where the loss of Bmp signaling alters endoderm morphogenesis, which sensitizes embryos to ethanol-induced CNCC apoptosis, leading to craniofacial defects. Collectively, our work continues to build on a model of Bmp-dependent ethanol susceptibility during craniofacial development, establishing and expanding on novel mechanistic concepts in gene–ethanol interactions for future studies in FASDs.

## 2. Materials and Methods

### 2.1. Zebrafish (Danio rerio) Care and Use

Zebrafish embryos were raised and cared for using IACUC protocols approved by the University of Louisville. Adult fish were maintained at 28.5 °C with a 14/10 h light/dark cycle. The *bmp4^st72^* [22], *smad5^b1100^* [10], *sox17:EGFP^s870^* [23], and *sox10:EGFP^ba2^* [24] zebrafish lines were previously described.

### 2.2. Zebrafish Staging and Ethanol Treatment

Eggs from random heterozygous crosses were collected, and embryos were morphologically staged [20], sorted into sample groups of 100, and reared at 28.5 °C to the desired developmental time points. All groups were incubated in embryo media (EM). At 10 hpf, EM was changed to either fresh EM or EM containing 1% ethanol (*v*/*v*) and was washed out with 3 fresh changes in EM at 18 hpf. This dose of ethanol was chosen as it is the highest dose that does not cause craniofacial defects in wild-type embryos [5,25,26,27,28,29]. This dose of ethanol equilibrates within 5 min of exposure to an average ethanol tissue concentration of 50 mM (approximately 30% of the media), which is roughly equivalent to a human blood alcohol concentration of 0.23. While a binge dose, it is physiologically relevant to FASDs, with humans readily surpassing this amount [6,21,29,30,31,32,33,34].

### 2.3. Hybridization Chain Reaction (HCR) and Immunofluorescence

Embryos were collected at 18 hpf, dechorionated, and fixed in 4% paraformaldehyde/PBS overnight at 4 °C. HCR protocol was previously described [35]. HCR amplifiers and buffers were acquired from Molecular Instruments (Los Angeles, CA, USA). HCR probes against *nkx2.3* were designed as previously described [36]. Immunofluorescence was performed as previously described [12]. Primary antibody against cleaved Caspase-3 (#9661, Cell Signaling, Danvers, MA, USA) was used at 1/200. A secondary antibody, Alexa Fluor anti-rabbit 568, was used at 1/500. Confocal images were taken using an Olympus FV1000 or an Olympus FV3000 microscope (Olympus, Center Valley, PA, USA).

### 2.4. Pouch Measurements

Linear measures of the pouches were obtained in FIJI (version 1.54k) [37] from 36 hpf embryos. The length of the pouch was defined as the distance from the most dorsal initial branching point from the medial endoderm to the most ventral end of the out-pocketing endoderm. The width of the pouch was reported as the average of three measurements in the medial portion of each pouch, equidistant from the most dorsal and most ventral end of each pouch. Pouch depth was determined using an orthogonal view (*Z*-axis) of each pouch and recorded from where the medial endoderm is no longer visible to the most lateral tip of the pouch. The number of slices and their thickness (in µm) was added to give the measure of pouch depth. As a result, the measures of pouch depth were more stratified compared to the length and width. We measured pouches 1–4 individually, but pouches 5 and 6 were not clearly separated and were measured as a combined single pouch. Volumetric measurements were obtained using FIJI. From each endoderm-labeled confocal stack, we generated a sub-stack for each pouch defining the stack range from where it no longer connects to the medial endoderm to the most lateral tip of the pouch. The image threshold function was then used to determine and record the lower and upper fluorescent threshold of the pixels in the sub-stack. Three-dimensional ROI Manager was then used to track the continuous pixels present in each sub-stack. From this, the volume value was measured and recorded as pixel volume (voxel), then converted into microns.

### 2.5. Analysis of Volumetric Fluorescent Intensity Measurements and Co-Labeling of Apoptosis and CNCCs

Volumetric fluorescent intensity is measured by selecting two volumetric entities as described above, and the colocalization intensity (white part) is integrated in the 3D Manager in FIJI, giving a fluorescent intensity. For the caspase/apoptosis experiments, the number of co-labeled puncta (white particles) of Caspase (apoptotic) and CNCCs (*sox10:eGFP*) was manually counted.

### 2.6. Morpholino Injection and Cartilage Staining

Wild-type or *bmp4* mutant embryos were injected with ~10 ng of either standard control or translation blocking *nkx2.*3-MO (Gene Tools) at the 1- to 2-cell stage and raised to 5 dpf. This dose and the resulting phenotypes were consistent with previously a published study on *nkx2.*3-MO [19] and at a dose below the toxicity threshold of 12 ng [38]. At 5 dpf, zebrafish larva were fixed, and facial cartilages were stained with alcian blue [39]. Whole-mount, ventral view, brightfield images of the viscerocranium were taken on an Olympus BX53 compound microscope (Olympus, Center Valley, PA, USA).

### 2.7. Statistical Analyses

To determine the sample size, power analyses were performed for all experiments based on preliminary or published data with α = 0.05 at 80% power with an effective size between 0.3 and 0.4. All experiments were performed in triplicate. The angle of the ceratohyal (AOC) of *nkx2.3-MO* in the wild type, endoderm measures, and cell counts of apoptotic CNCCs were analyzed with a two-way ANOVA (type III) with Tukey’s multiple comparisons test comparing effects between genotype and treatment. The AOC of *nkx2.3-MO* in *bmp4* mutants was analyzed with a 3-way ANOVA (type III) with Tukey’s multiple comparisons test comparing effects between genotype, morpholinos, and treatment using Graphpad Prism 10.2.3 (Graphpad Software Inc., La Jolla, CA, USA).

## 3. Results

### 3.1. Pharyngeal Pouches Are Malformed in Ethanol-Treated Bmp Mutants

**Figure 1:**
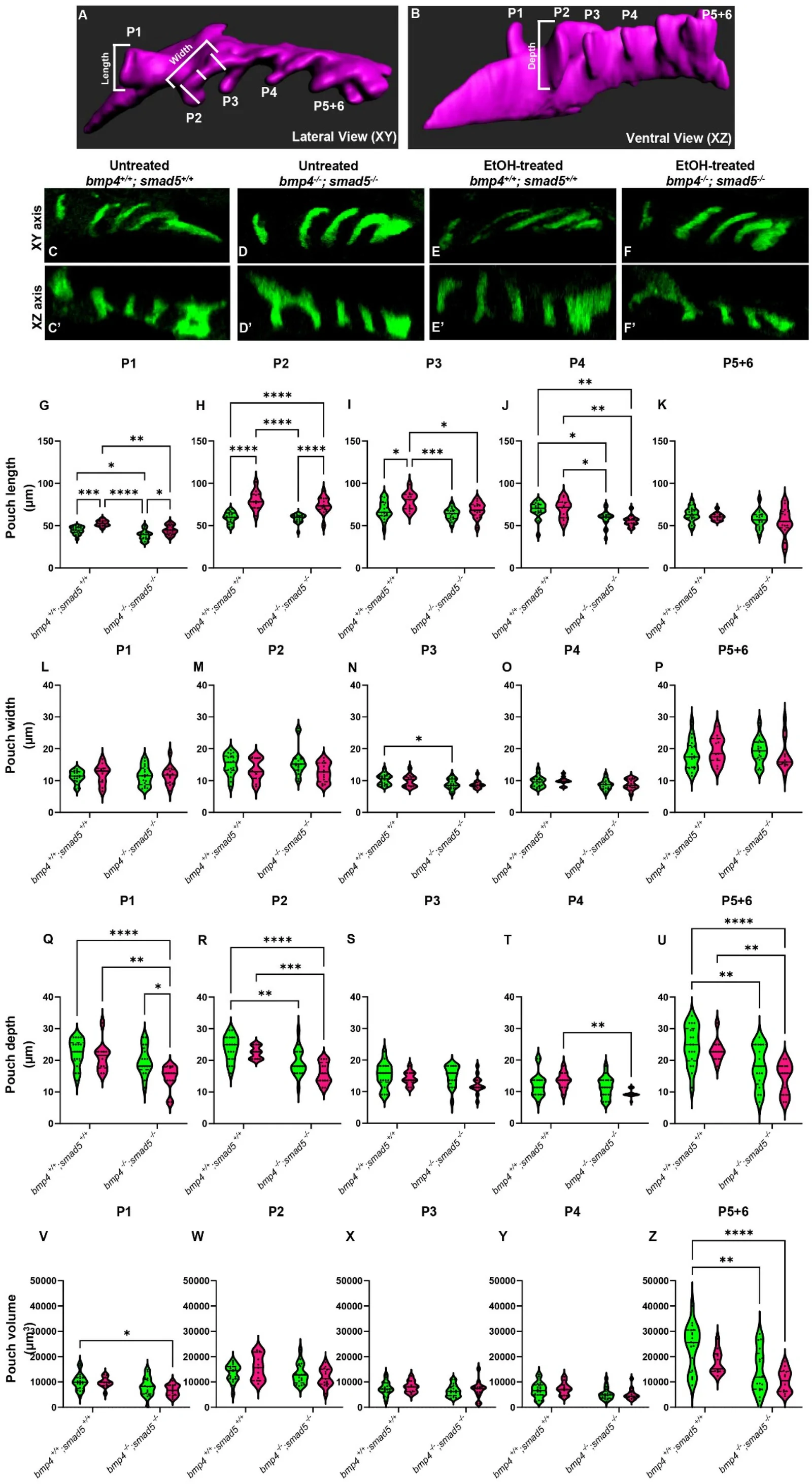
Ethanol alters the shape and size of the pharyngeal pouches. (**A**,**B**) Schematic representations of pharyngeal endoderm at 36 hpf (A = lateral [XY] view; B = ventral [XZ] view). Pouches 1–4 are shown, and pouches 5 and 6 have yet to fully separate and are counted as a single pouch (5 + 6). The length was measured from the start to the end of the out-pocketing endoderm (length of pouch 1, **panel A**). The width of the pouch was an average of three measures in the medial portion of that specified pouch (width of pouch 2, **panel A**). Z–depth was determined using the orthogonal view (XZ) and recorded from the stack where the medial endoderm is not visible to the tip of the pouch (depth of pouch 2, **panel B**). (**C**–**F′**) Representative confocal images at 36 hpf of untreated and ethanol-treated wild-type and *bmp4*; *smad5* double mutant embryos showing the pharyngeal endoderm labeled with *sox17:eGPF* (lateral view, anterior to the left). (**G**–**U**) Linear pouch measurements, length (**G**–**K**), width (**L**–**P**), and depth (**Q**–**U**) of each pharyngeal pouch. (**V**–**Z**) Measures of pouch volume. *: *p* ≤ 0.05. **: *p* ≤ 0.01. Every additional “*” beyond two reduces the *p* value by a factor of 10.

We have previously shown that mutations in Bmp signaling components sensitize zebrafish embryos to ethanol-induced jaw malformations through increases in the size of the anterior pharyngeal endoderm [20]. Defects in anterior endoderm development are known to impact jaw development but not development of the posterior endoderm cartilage elements, while defects in pouch morphogenesis disrupt the formation of the posterior cartilage elements [12,16]. Our work demonstrated that ethanol broadly disrupts the development of the craniofacial skeleton, both the jaw and posterior cartilage elements in ethanol-treated Bmp mutants [20], suggesting that Bmp–ethanol interactions are disrupting pouch morphogenesis in addition to defects to anterior endoderm morphogenesis. Similarly to the anterior endoderm, we observed that while the pouches largely form in both untreated and ethanol-treated wild types and *bmp4*; *smad5* double-mutant embryos, their shape and size may be altered (Figure 1C–F′).

To test this, we analyzed the length, width, z-depth, and volume of the pharyngeal pouches in ethanol-treated *bmp4*; *smad5* double-mutant embryos at 36 hpf. We have previously shown that this double-mutant line is 100% penetrant for highly expressive ethanol-induced defects to the facial skeleton formation [20]. Pouch length was defined as the distance from the dorsal-most end to the ventral-most end of the pouch (Figure 1A, length); pouch width was the average of three measures equidistant from the pouch ends (Figure 1A, width); and the z-depth was determined using the orthogonal view and recorded as the thickness of each z-slice from where the medial endoderm is not visible to the tip of the pouch (Figure 1B, depth). We measured pouches 1–4 individually, but pouches 5 and 6 were not clearly separated and were measured as a combined single pouch.

Overall, pouch formation was not largely disrupted due to genotype or treatment at 18 (at the end of ethanol treatment), 24, 30, or 36 hpf (Figure 1C–F′; Supplementary Figure S1A–L). However, while variable between the pouches, we did find significant changes in pouch length and depth, while pouch width was largely unaffected at 36 hpf (Figure 1G–U). Pouches 1, 2, and 3 were significantly elongated by genotype, ethanol treatment, and genotype by ethanol treatment (Figure 1G–K). Pouch 4 showed only significant elongation due to genotype, and pouch 5 + 6 was largely unaffected (Figure 1L–P). For pouch depth, pouch 1 showed a significant reduction in depth in ethanol-treated *bmp4*; *smad5* double mutants compared to the untreated wild type and *bmp4*; *smad5* double mutant siblings and ethanol-treated wild-type siblings (Figure 1Q). Pouches 2 and 5 + 6 show a reduction in depth in Bmp mutants compared to wild-type siblings, independent from treatment (Figure 1R,U). Pouches 3 and 4 were largely unaffected (Figure 1S,T). Finally, measuring the volume of each individual pouch, only pouches 1 and 5 + 6 show a significant reduction in volume in ethanol-treated mutants (Figure 1V,Z). Overall, these measurements indicate that while the pouches largely form, Bmp–ethanol interactions alter the size and shape of the pouches, suggesting that these changes in pouch shape and size may be impacting facial development in ethanol-treated *bmp4*; *smad5* double mutants.

### 3.2. Expression of nkx2.3 Is Reduced in Bmp Mutants but Not Impacted by Ethanol

**Figure 2:**
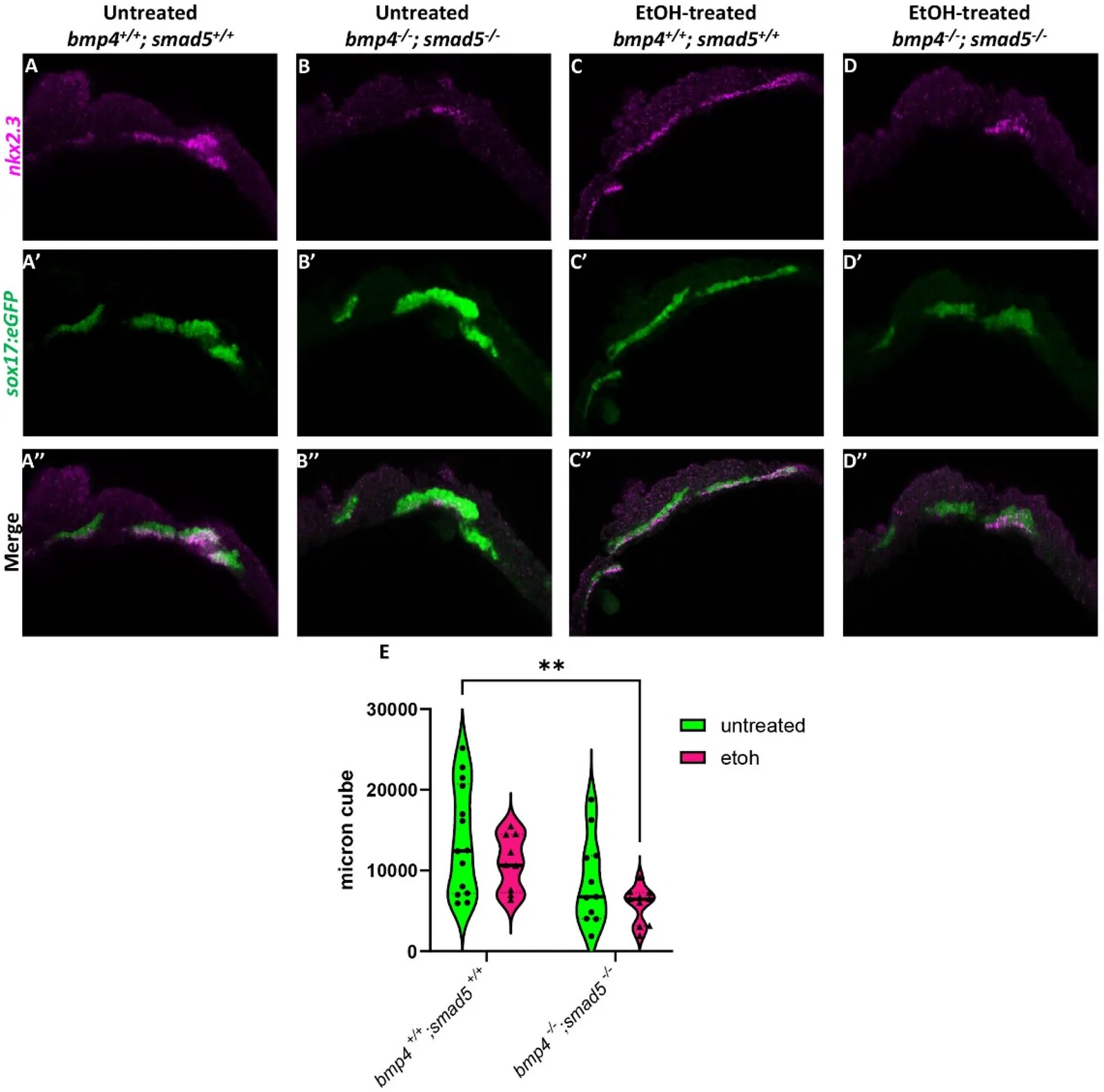
Ethanol does not alter *nkx2.3* expression in Bmp mutants. (**A**–**D″**) Confocal images of *sox17:eGPF* labeled endoderm (green) and *nkx2.3* mRNA (magenta) expression using HCR at 18 hpf (lateral views, anterior to the left). (**E**) Quantification of volumetric fluorescent intensity measurements of *nkx2.3* gene expression and *sox17:eGFP* labeling. Solid lines indicate the median of that treatment group. Dashed lines show 95% confidence of the median. **: *p* ≤ 0.01.

We have previously shown that ethanol does not attenuate Bmp signaling [20], suggesting that ethanol may be interacting with downstream Bmp targets to disrupt craniofacial development. A study by Li et al. (2019) showed that *nkx2.3* is a downstream target of Bmp signaling regulating pouch specification [17]. To test whether ethanol affects target gene expression downstream of Bmp signaling, we used a hybridization chain reaction (HCR) to assess the expression of *nkx2.3* at 18 hpf in wild-type and *bmp4*; *smad5* double mutant embryos with the endoderm labeled with the *sox17:eGFP* transgene. At 18 hpf, *nkx2.3* is expressed most in the developing pouch and ethanol does not reduce this expression (Figure 2A,C). Bmp mutant embryos show reduced *nkx2.3* expression regardless of ethanol (Figure 2B,D). Volumetric fluorescent intensity measurements of colocalized fluorescence of *nkx2.3* and *sox17:eGFP* expression in the pouches of untreated and ethanol-treated wild-type and *bmp4*; *smad5* double mutant embryos were 13,781, 10,974, 8646, and 5726 µm^3^, respectively (Figure 2E). Despite the statistically significant differences between untreated wild-type and ethanol-treated *bmp4*; *smad5* double mutant embryos observed in *nkx2.3* colocalization intensity in the pouches, our data demonstrate that ethanol does not directly attenuate *nkx2.3* expression in the endoderm in wild-type or *bmp4*; *smad5* double mutant embryos (Figure 2E). Overall, these data overall suggest that ethanol does not attenuate Bmp target gene expression at the level of transcription.

### 3.3. Ethanol Does Not Sensitize nkx2.3 Morphants to Craniofacial Defects 

**Figure 3:**
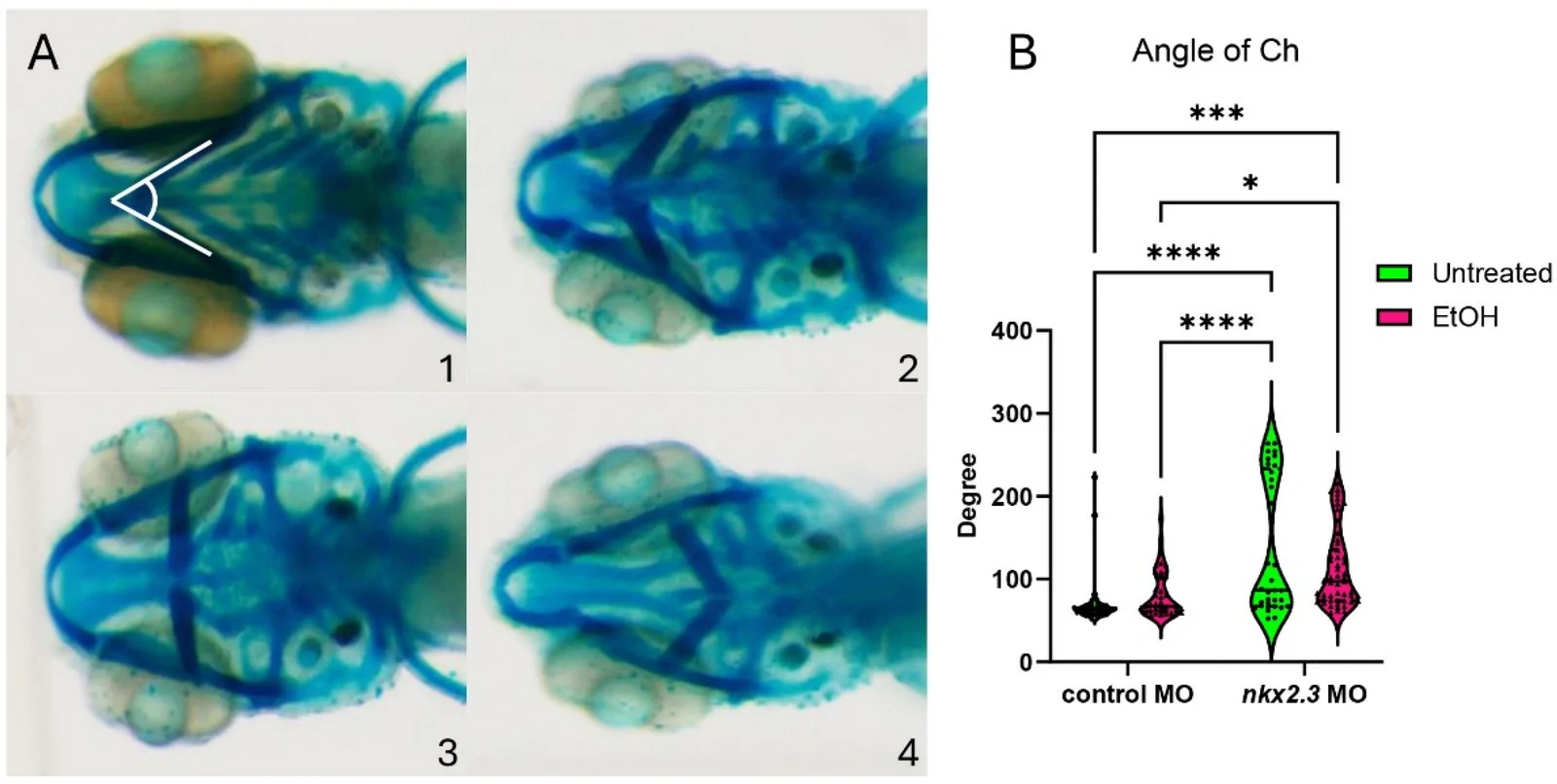
Ethanol does not exacerbate facial defects in *nkx2.3* morphants. (**A**, **1**–**4**) The 5 dpf alcian-stained standard control MO and *nkx2.3* MO-injected larvae (~10 ng; ventral views, anterior to the left, white lines in panel 1 represent angle of ceratohyal (AOC) measures). Four categories were used to assess the severity of facial defects. Category #1 = AOC < 90°. Category #2 = AOE from 90 to 135°. Category #3 = 135 and 180. Category #4 = inverted ceratohyal (AOC > 180). Additional representative images are provided in Supplementary Figure S2. (**B**) Quantification of the AOC in untreated and ethanol-treated standard control MO and *nkx2.3* MO-injected larvae. Solid lines indicate the median of that treatment group. Dashed lines show 95% confidence of the median. *: *p* ≤ 0.05. ***: *p* ≤ 0.001. ****: *p* ≤ 0.0001.

While ethanol does not decrease *nkx2.3* gene expression, it may still interact with the *nkx2.3* gene product. The loss of *nkx2.3* does lead to defects in the posterior cartilage elements [19]. To test if *nkx2.3* knockdown sensitizes embryos to ethanol-induced facial defects, we injected wild-type embryos with *nkx2.3* translation-blocking morpholinos and treated them with ethanol from 10 to 18 hpf when *nkx2.3* is required for endoderm specification [17]. Knockdown of *nkx2.3* resulted in a range of phenotypes from the loss of the ceratobranchial cartilages to the inversion of the ceratohyal cartilages. We categorize these phenotypes based on the severity of the angle of ceratohyal (AOC) (Figure 3A, 1–4; Supplementary Figure S2A–D″). Category 1 is the wild type in phenotype with the AOC less than 90°. Category 2 includes the mild phenotype of AOC being increased compared to wild-type larvae, ranging from 90 to 135°. Category 3 comprises larvae with the AOC ranging from 135 to 180°. Finally, category 4 consists of larvae where the AOC is more than 180° (Figure 3A, 1–4). Control MO-injected larvae are largely phenotypically normal, though ethanol treatment does increase phenotype category 2 over category 1 (95.12% vs. 74.36%; Table 1). However, measures of the AOC are not significantly different between untreated and ethanol-treated control MO larvae (Figure 3B). Untreated larvae injected with *nkx2.3* MO have a lower percentage of larvae in category 1, at 52.78%, and an increased percentage in other categories as follows: category 2: 8.3% and category 4: 38.89% (Table 1). It also shows a significant increase in the AOC compared to untreated and ethanol-treated control MO-injected larvae (Figure 3B). Ethanol does not increase the penetrance of facial defects in *nkx2.3* morphants compared untreated *nkx2.3* morphants (Figure 3B, Table 1), indicating that *nkx2.3*-MO larvae are insensitive to ethanol.

Table 1 Penetrance and expressivity of *nkx2.3* MO-injected wild-type larvae in ethanol. Percent of larvae, either standard control MO or *nkx2.3* MO-injected, that show the phenotypes in Figure 3 for each treatment group. The numbers in parentheses in different phenotype categories show the number of larvae overall and those exhibiting each phenotype category.

### 3.4. Ethanol Does Not Exacerbate Facial Defects bmp4 Mutants with nkx2.3 MO

**Figure 4:**
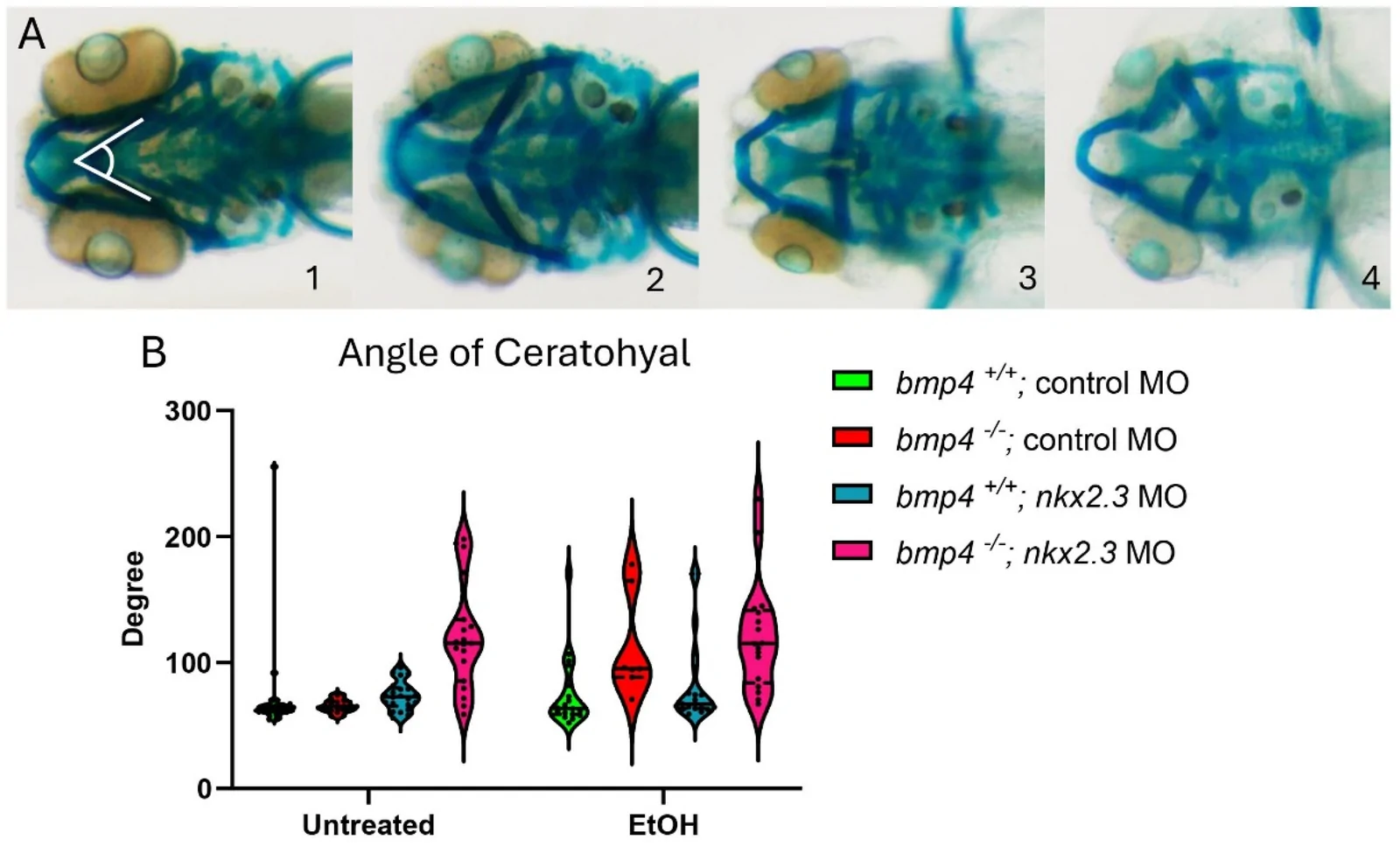
Knockdown of *nkx2.3* does not further sensitize the in *bmp4* mutant larvae to ethanol. (**A**, **1**–**4**) The 5 dpf alcian-stained larvae untreated and ethanol-treated, standard control MO and *nkx2.3* MO-injected (~10 ng) wild-type and *bmp4* mutant larvae (ventral view, anterior to the left, white lines in panel 1 represent angle of ceratohyal (AOC) measures). Four categories were used to assess the severity of facial defects. Category #1 = AOC < 90°. Category #2 = AOE from 90 to 135°. Category #3 = 135 and 180. Category #4 = inverted ceratohyal (AOC > 180). Additional representative images are provided in Supplementary Figure S2. (**B**) Quantification of the AOC of untreated and ethanol-treated standard control MO and *nkx2.3* MO-injected larvae. (**B**) Quantification of the AOC in untreated and ethanol-treated standard control MO and *nkx2.3* MO-injected wild-type and *bmp4* mutant larvae. Solid lines indicate the median of that treatment group. Individual graph statistics are shown in Supplementary Table S1.

Larvae lacking *bmp4* are ethanol-sensitive and exhibit facial defects [20]. We hypothesized that knocking down *nkx2.3* in *bmp4* mutant larvae would further sensitize these larvae to ethanol, exacerbating their ethanol-induced phenotypes closer to the *bmp4*; *smad5* double-mutant and DM-treated larvae [12,20]. As described in Figure 3, we categorized the facial phenotypes of wild-type and *bmp4* mutant siblings into four groups based on the AOC and presence of facial defects. Untreated control MO-injected wild-type and *bmp4* mutant larvae do not display facial defects (Figure 4A, 1–4, Table 2, Supplementary Table S1). The AOC of ethanol-treated control MO-injected *bmp4* mutant larvae did show a significant increase in the AOC compared to untreated control MO larvae (Figure 4B, Table 2, Supplementary Table S1). These results are consistent with our previously published results on *bmp4*–ethanol interactions on viscerocranial development [20]. Morpholino knockdown of *nkx2.3* in untreated and ethanol-treated wild-type larvae shift categories, as observed in Figure 3, though the AOC is not significantly different (93.33% to 81.82% in category 1 and 3.33% to 18.18% in category 2; Table 2, Supplementary Table S1). However, knockdown of *nkx2.3* in *bmp4* mutant larvae significantly shifts the phenotype categories compared to untreated control MO- and *nkx2.3* MO-injected wild types (Figure 4B, Table 2, Supplementary Table S1). However, ethanol does not increase the AOC in *nkx2.3* MO-injected *bmp4* mutant larvae compared to control MO-injected *bmp4* mutant larvae (Figure 4B, Table 2, Supplementary Table S1). These data indicate that while knocking down *nkx2.3* in untreated *bmp4* mutant larvae does mildly exacerbate the facial defects, *nkx2.3* knockdown does not sensitize *bmp4* mutant larvae to ethanol-induced facial defects, suggesting that ethanol is acting independent of Bmp signaling to disrupt facial development.

Table 2 Penetrance and expressivity of *nkx2.3* MO-injected wild-type and *bmp4* mutant larvae in ethanol. Percent of standard control MO or *nkx2.3* MO and *nkx2.3* MO-injected wild-type or *bmp4* mutant larvae that show the phenotypes in Figure 4 for each treatment group. The numbers in parentheses in different phenotype categories show the number of larvae overall and those exhibiting each phenotype category.

### 3.5. Ethanol Sensitizes Bmp Mutants to Increased CNCC Apoptosis

**Figure 5:**
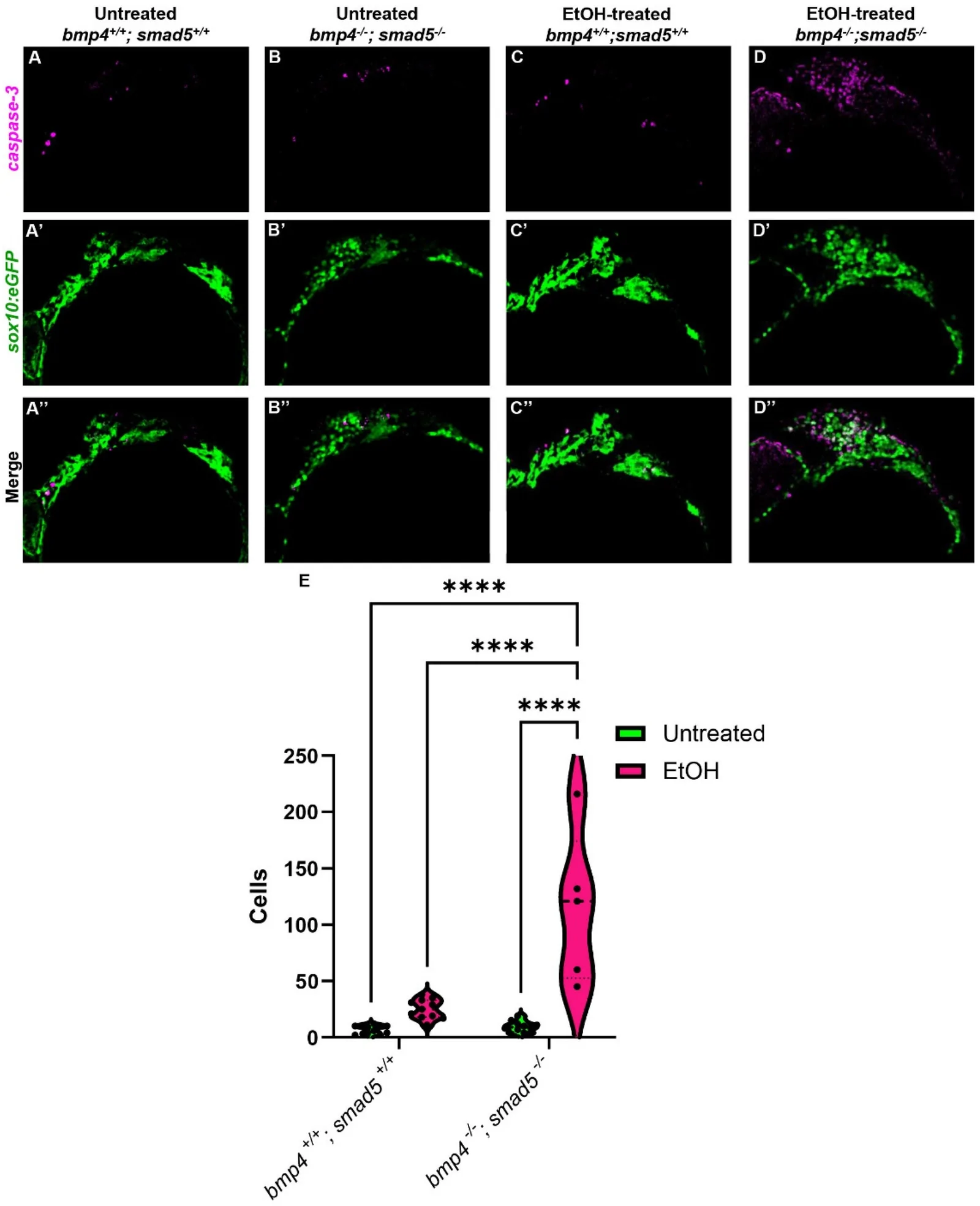
Ethanol-treated Bmp mutants have elevated CNCC apoptosis. (**A**–**D″**) Confocal images of CNCCs (*sox10:eGFP*) and cleaved Caspase-3 at 18 hpf (lateral view, anterior to the left). (**E**) Quantification of manually counted detectable apoptotic CNCCs in untreated and ethanol-treated wild-type and *bmp4*; *smad5* double mutant embryos. Solid lines indicate the median of that treatment group. Dashed lines show 95% confidence of the median. ****: *p* ≤ 0.0001.

The endoderm is critical in multiple aspects of craniofacial development, particularly CNCC survival [40,41]. In embryos that completely lack an endoderm, SRY-box transcription factor 32 (*sox32*) mutants, CNCCs migrate and condense in the pharyngeal arches but undergo apoptosis, contributing to an almost complete loss of the facial skeleton [40]. In mutants in endoderm development where the endoderm is present but fails to form properly, CNCC death is still observed [14,42]. In addition, multiple studies have shown that ethanol also induces CNCC apoptosis in different animal models [27,43,44,45], suggesting that ethanol-treated Bmp mutants will have increased CNCC apoptosis. To test that ethanol exacerbates CNCC apoptosis in Bmp mutants, we performed immunostaining against cleaved Caspase-3 on untreated and ethanol-treated wild-type and *bmp4*; *smad5* double mutant embryos, labeling the CNCC with *sox10:eGFP*. The number of apoptotic CNCCs in untreated wild-type embryos is low and contained mostly in the anterior first arch (Figure 5A–A″). In the untreated *bmp4*; *smad5* double mutant and ethanol-treated wild-type embryos, apoptotic CNCCs expand to the posterior of arch 1 and into arch 2 (Figure 5B–C″). The number of apoptotic CNCCs in the untreated wild-type and *bmp4*; *smad5* double mutant embryos is relatively low, with mean values of 7.18 and 9.27 cells, respectively, while the number of apoptotic cells in ethanol-treated wild-type embryos increases to a mean of 25.18 cells (Figure 5E). However, the average number of apoptotic CNCCs in ethanol-treated *bmp4*; *smad5* double mutants is 114.8 cells, significantly increased compared to all other groups and distributed evenly throughout most of arches 1 and 2 (Figure 5D). This increase in cell apoptosis in ethanol-treated *bmp4*; *smad5* double mutants persists and is observed at 24 and 30 hpf, though the overall number of apoptotic cells is decreased compared to 18 hpf (Supplementary Figure S3A–H″). Overall, these data indicate that mutations in the Bmp pathway sensitize CNCCs to ethanol-induced apoptosis and suggest that Bmp–ethanol interactions are disrupting signaling from the endoderm to the CNCC. Based on this, we hypothesize a two-hit model, where Bmp mutation in combination with ethanol exposure disrupt endoderm morphogenesis, altering signaling events between the endoderm and CNCCs, leading to increases in CNCC apoptosis and subsequent defects in the facial skeleton.

## 4. Discussion

Pouch formation is critical to the overall development of the craniofacial skeleton, with disruptions to pouch out-pocketing leading to the malformation and/or loss of viscerocranial cartilage elements in the facial skeleton [12,13,14]. Multiple pathways are required to regulate several key aspects of pouch formation, including the Bmp and Fgf signaling pathways [12,13,14]. Bmp signaling appears to lie at the head of this regulation, driving both pouch out-pocketing via the expression of *fgfr4* and pouch specification via *nkx2.3* [12,17]. The timing of *nkx2.3* gene expression and Fgf signaling responses in wild-type and Bmp knockdown suggest that *nkx2.3* is upstream of Fgf in pouch development [12,17]. This suggests that Bmp signaling is a key regulator in pouch morphogenesis, with the loss of Bmp signaling leading to a reduced expression of multiple downstream target genes. We have recently shown that mutations in multiple Bmp signaling components sensitize embryos to ethanol-induced defects throughout the viscerocranium [20]. We found that these defects are generated in part by disrupting anterior endoderm morphogenesis [20]. However, while the anterior endoderm is necessary for jaw development, it does not contribute to defects in the posterior cartilage elements [16]. Given that we observed posterior cartilage defects in our ethanol-treated Bmp mutants, this suggests that pouch morphogenesis was also impacted by these Bmp–ethanol interactions. Here, we showed that the ethanol-induced defects in the facial skeleton in Bmp mutants are independent of the Bmp-*nkx2.3* signaling pathway. While we showed ethanol-induced changes in pouch morphology, we showed *nkx2.3* expression is not impacted by ethanol exposure and that knocking down *nkx2.3* does not further sensitize wild-type nor *bmp4* mutants to ethanol-induced facial defects. Strikingly, we observed increased cell apoptosis in CNCCs in ethanol-treated Bmp mutants, suggesting a two-hit model where ethanol sensitizes Bmp mutants to CNCC death, leading to facial defects.

### 4.1. Ethanol Induces Cell Death in Bmp Mutants

Our previous study has shown that blocking Bmp signaling does not alter endoderm cell numbers [12]. Yet, our data here show changes in pouch shape but not the overall volume, suggesting that ethanol is not changing the number of endoderm cells in any meaningful way. In our previous study, we saw a loss of Bmp signaling responses in the endoderm in Bmp mutants regardless of ethanol treatment, while we did not observe any changes to Bmp signaling responses in non-endoderm tissues in either Bmp mutants or due to ethanol exposure [20]. This demonstrates that ethanol does not directly impinge on Bmp signaling activity, raising the question of the mode of action of ethanol on facial development. Multiple lines of evidence show that ethanol induces cell death in the CNCC [27,43,44,45]. Our data show that our Bmp–ethanol interactions significantly increase cell apoptosis in migrating CNCCs. However, we only observed this increase in ethanol-treated Bmp mutants, not in ethanol-treated wild-type or untreated Bmp mutant larvae alone, suggesting a two-hit model where Bmp mutation sensitizes embryos to ethanol-induced cell death in the CNCC. Multiple mechanisms that include increased oxidative stress, changes in calcium flux, and changes in gene expression [46,47] could lie at the heart of our Bmp–ethanol interactions. Multiple models have shown that changes in ethanol metabolism, either directly through alcohol dehydrogenase/aldehyde dehydrogenase variants or indirectly through the Fanconi anemia group D2 (Fancd2) DNA repair enzyme, decrease ethanol metabolism and increase ROS accumulation and DNA damage, leading to increased cell death. Expanding beyond ethanol metabolism, ethanol-induced changes in intracellular calcium levels directly contribute to increased cell death by regulation gene expression in CNCCs [21,43]. Studies in chicks and zebrafish show that increased calcium levels decrease Wnt/β-catenin signaling, altering gene expression and increasing apoptosis, providing yet more experimental targets for Bmp epistasis [47]. Future tests with a calcium chelator would determine whether the manipulation of calcium levels is protective against ethanol-induced facial defects in Bmp mutants.

While zebrafish is a strong model for humans, limitations do exist in ethanol metabolism studies. Zebrafish appear able to tolerate higher tissue levels of ethanol that broadly surpass blood alcohol concentrations (BACs) in humans. However, due to their nature, these analyses cannot be directly compared. It is also possible that anatomical differences may impact zebrafish differently than mammals [48]. Ethanol can be absorbed by zebrafish through the skin and gills, while mammals require ingestion. This could result in a difference in metabolic machinery that metabolizes ethanol between zebrafish and mammals. Ultimately, future work will be needed to determine the mechanism underlying CNCC death in our ethanol-sensitive Bmp mutants and how this relates to humans.

While the downstream Bmp target *nkx2.3* did not sensitize embryos to ethanol-induced defects, this does not exclude other pathways contributing to our ethanol-induced phenotypes. In *sox32* mutants that completely lack an endoderm, CNCCs properly migrate and condense in the pharyngeal arches but show increased CNCC death after condensation [40]. In mutants that still generate the endoderm but display defects in pouch size and shape, such as vestigial-like family member 2a (*vgll2a*), increased CNCC death in the arches is still observed [42]. This suggests that *vgll2a* could be a Bmp target in the endoderm that regulates CNCC apoptosis, though more work is needed to determine if *vgll2a* is a bonafide Bmp target and how it regulates CNCC survival. We have previously shown that mutations in platelet-derived growth factor receptor alpha (*pdgfra*) sensitize embryos to CNCC apoptosis, with ethanol acting downstream of *pdfgra* at the level of the mechanistic target of rapamycin kinase (mTOR), increasing apoptosis [27]. The upregulation of mTOR via exogenous L-leucine supplementation in *pdgfra* mutants was sufficient to reduce ethanol-induced cell death [27]. We have previously shown that the endoderm does express the Pdgf ligands, *pdgfaa* and *pdfgab*, suggesting that ethanol-treated Bmp mutants may have decreased Pdgf signaling that leads to increased apoptosis. It is possible that changes in mTOR-based cell survival may be disrupted in ethanol-treated Bmp mutants. However, this is unlikely, as L-leucine supplementation did not alleviate ethanol-induced facial defects in Bmp mutants. Combining these data implicates that CNCCs receive cell survival cues from the endoderm when in close proximity, either through direct cell–cell contact or close paracrine-based cell signaling.

We observed similar pouch shape changes in our ethanol-treated Bmp mutants; yet, unlike these previous examples, we observed CNCC apoptosis in migrating CNCCs some distance from the forming pouches. This suggests that the endoderm provides cell survival cues to the migrating CNCCs prior to completed pouch out-pocketing and CNCC condensation in the pharyngeal arches. However, the cell survival signals from the endoderm and how they are transmitted to the CNCCs remain unknown. Only two known endoderm-based Bmp-dependent targets have been identified and described, *fgfr4* and *nkx2.3* [12,17,19]. Our work here rules out *nkx2.3* in Bmp–ethanol interactions, and we are currently exploring Fgf signaling (described below). Future work will need to identify more Bmp targets and the pathways they may regulate to facilitate well-reasoned experimental paradigms to examine the Bmp-dependent signaling pathways driving ethanol-induced facial defects. We are currently embarking on single-cell transcriptomic approaches to both the endoderm and CNCCs that will help shed light on the Bmp-dependent ethanol-sensitive signaling events connecting endoderm morphogenesis to CNCC survival.

### 4.2. Ethanol-Sensitive Bmp-Dependent Pathway Regulating Pouch Development

We have previously shown that the loss of Bmp signaling results in severe morphological defects to the endoderm through the downstream target Fgf signaling [12]. A study by Li et al. in 2019 [17] added *nkx2.3* as another downstream target of Bmp signaling in pouch formation. More recent work from the same lab showed that the pouches largely form properly, though subtle shape changes can still be observed in *nxk2.3* morphants [19]. While not as severe, Bmp–ethanol interactions also disrupt endoderm morphogenesis, though ethanol does not act on Bmp signaling [20] or its downstream target of *nkx2.3* (this study). However, ethanol may still act on additional downstream targets of Bmp signaling beyond *nkx2.3*. It is possible that ethanol may be acting on Fgf signaling, leading to defects in endoderm morphogenesis. Fgf signaling acts as the migratory cue to the endoderm for pouch out-pocketing [8]. We have shown that Bmp signaling regulates the reception of Fgf signaling through the expression of *fgfr4* in the pre-pouch endoderm [12]. Although Fgf mutants have pouch defects similar to Bmp loss [12,17,19], our pilot screens show they are not ethanol-sensitive, though these results need to be confirmed. We are currently exploring the role of Fgf signaling and if the loss of Fgf signaling sensitizes Bmp mutants to ethanol-induced facial defects.

Another potential Bmp target in pouch development is the Wnt/planar cell polarity (Wnt/PCP) signaling pathway. Multiple Wnt ligands such as *wnt11r* and *wnt4a* have been shown to take part in the pouch morphogenesis through the destabilization and re-stabilization of points of cell adhesion necessary to promote pouch out-pocketing [13,14]. We have observed reductions in the expression of the Wnt/PCP receptor frizzled class receptor 8a (*fzd8a*) in the endoderm in DM-treated embryos, suggesting that Bmp signaling may be regulating the reception of Wnt/PCP signaling similar to its regulation of Fgf signaling [12]. Moreover, it has been shown that the Wnt/PCP pathway members glypican 4 (*gpc4*) and VANGL planar cell polarity protein 2 (*vang2*) interact with ethanol, disrupting craniofacial development [49]. This suggests that the Wnt/PCP pathway components may be downstream ethanol-sensitive targets in Bmp mutants. It is also possible that more than one of these downstream Bmp targets may be driving ethanol sensitivity in Bmp mutants and future work will be needed to dissect all potential interactions between Bmp, Fgf, Wnt/PCP, and ethanol during endoderm morphogenesis.

In conclusion, our results show that zebrafish provide a valuable model system for determining ethanol-sensitive tissue events that contribute to facial defects in FASDs. Given the genetic and developmental homology between zebrafish and humans, the zebrafish provides a powerful model to examine complex genetic signaling pathways and generate a deeper mechanistic understanding of ethanol interactions with these pathways on craniofacial development. Here, we show that ethanol alters endodermal pouch morphology and subsequent facial development in ethanol-treated Bmp mutants independent of *nxk2.3*, resulting in increased CNCC apoptosis. This suggests a two-hit model where the loss of Bmp signaling alters endoderm morphology, which sensitizes embryos to ethanol-induced CNCC apoptosis, leading to craniofacial defects. Thus, our work provides a novel mechanistic understanding of gene–ethanol interactions related to the complex signaling and tissue interactions driving craniofacial development. Ultimately, this will provide a conceptual framework and a mechanistic paradigm of ethanol-induced birth defects and connect ethanol exposure with concrete cellular events that could be sensitive beyond facial development.

## Figures and Tables

**Table 1:** Ethanol-*nkx2.3* penetrance and expressivity.

		Phenotype Categories			
		1	2	3	4
Untreated	Control MO (41)	95.12% (39)	0	2.44% (1)	2.44% (1)
	*nkx2.3* MO (36)	52.78% (19)	8.3% (3)	0	38.89% (14)
1% EtOH	Control MO (39)	74.36% (29)	20.51% (8)	5.13% (2)	0
	*nkx2.3* MO (53)	47.17 (25)	22.64% (12)	9.43% (5)	20.75% (11)

**Table 2:** Ethanol–*nkx2.3–bmp4* penetrance and expressivity.

			Phenotype Categories			
			1	2	3	4
Untreated	Control MO (41)	*bmp4^+/+^ (30)*	93.33% (30)	3.33% (1)	0	3.33% (1)
		*bmp4^−/−^ (18)*	100% (18)	0	0	0
	*nkx2.3* MO (36)	*bmp4^+/+^ (22)*	81.82% (18)	18.18% (4)	0	0
		*bmp4^−/−^ (19)*	26.31% (5)	47.37% (9)	0	26.31% (5)
1% EtOH	Control MO (39)	*bmp4^+/+^ (19)*	73.68% (14)	15.79% (3)	5.26% (1)	5.26% (1)
		*bmp4^−/−^ (8)*	25% (2)	37.5% (3)	25% (2)	12.5% (1)
	*nkx2.3* MO (53)	*bmp4^+/+^ (20)*	75% (15)	15% (3)	5% (1)	5% (1)
		*bmp4^−/−^ (17)*	29.41% (5)	41.18% (7)	17.65% (3)	11.76% (2)

## Data Availability

The original contributions presented in this study are included in the article/Supplementary Materials. Further inquiries can be directed to the corresponding author.

## Supplementary Materials

The following supporting information can be downloaded at: https://www.mdpi.com/article/10.3390/biomedicines13030755/s1, Figure S1: Confocal images of *sox17:EGFP*-labeled endodermal pouches in untreated and EtOH-treated wild type and *bmp4*; *smad5* double mutant embryos; Figure S2: Representative images of phenotypic categories in *nkx2.3*-MO injected larvae; Figure S3: CNCC apoptosis persists to 30 hpf in ethanol-treated Bmp mutants; Table S1: Ethanol-*bmp4-nkx2.3* three-way ANOVA statistics for Figure 4.

## References

[1] Williams J.F., Smith V.C. (2015). The Committee on Substance Abuse Fetal Alcohol Spectrum Disorders. Pediatrics.

[2] Fernandes Y., Lovely C.B. (2021). Zebrafish Models of Fetal Alcohol Spectrum Disorders. Genesis.

[3] Astley Hemingway S.J., Bledsoe J.M., Brooks A., Davies J.K., Jirikowic T., Olson E.M., Thorne J.C. (2018). Twin Study Confirms Virtually Identical Prenatal Alcohol Exposures Can Lead to Markedly Different Fetal Alcohol Spectrum Disorder Outcomes-Fetal Genetics Influences Fetal Vulnerability. Adv. Pediatr. Res..

[4] Hong M., Krauss R.S. (2012). Cdon Mutation and Fetal Ethanol Exposure Synergize to Produce Midline Signaling Defects and Holoprosencephaly Spectrum Disorders in Mice. PLoS Genet..

[5] Swartz M.E., Wells M.B., Griffin M., McCarthy N., Lovely C.B., McGurk P., Rozacky J., Eberhart J.K. (2014). A Screen of Zebrafish Mutants Identifies Ethanol-Sensitive Genetic Loci. Alcohol. Clin. Exp. Res..

[6] Zhang C., Ojiaku P., Cole G.J. (2013). Forebrain and Hindbrain Development in Zebrafish Is Sensitive to Ethanol Exposure Involving Agrin, Fgf, and Sonic Hedgehog Function. Birth Defects Res. Part A Clin. Mol. Teratol..

[7] Lovely C.B. (2020). Animal Models of Gene–Alcohol Interactions. Birth Defects Res..

[8] Raterman S.T., Metz J.R., Wagener F.A.D.T.G., Von den Hoff J.W. (2020). Zebrafish Models of Craniofacial Malformations: Interactions of Environmental Factors. Front. Cell Dev. Biol..

[9] Howe K., Clark M.D., Torroja C.F., Torrance J., Berthelot C., Muffato M., Collins J.E., Humphray S., McLaren K., Matthews L. (2013). The Zebrafish Reference Genome Sequence and Its Relationship to the Human Genome. Nature.

[10] Swartz M.E., Sheehan-Rooney K., Dixon M.J., Eberhart J.K. (2011). Examination of a Palatogenic Gene Program in Zebrafish. Dev. Dyn..

[11] Marelli F., Rurale G., Persani L. (2021). From Endoderm to Progenitors: An Update on the Early Steps of Thyroid Morphogenesis in the Zebrafish. Front. Endocrinol..

[12] Lovely C.B., Swartz M.E., McCarthy N., Norrie J.L., Eberhart J.K. (2016). Bmp Signaling Mediates Endoderm Pouch Morphogenesis by Regulating Fgf Signaling in Zebrafish. Development.

[13] Choe C.P., Collazo A., Trinh L.A., Pan L., Moens C.B., Crump J.G. (2013). Wnt-Dependent Epithelial Transitions Drive Pharyngeal Pouch Formation. Dev. Cell.

[14] Choe C.P., Crump J.G. (2014). Tbx1 Controls the Morphogenesis of Pharyngeal Pouch Epithelia through Mesodermal Wnt11r and Fgf8a. Development.

[15] Graham A., Richardson J. (2012). Developmental and Evolutionary Origins of the Pharyngeal Apparatus. EvoDevo.

[16] Balczerski B., Matsutani M., Castillo P., Osborne N., Stainier D.Y.R., Crump J.G. (2012). Analysis of Sphingosine-1-Phosphate Signaling Mutants Reveals Endodermal Requirements for the Growth but Not Dorsoventral Patterning of Jaw Skeletal Precursors. Dev. Biol..

[17] Li L., Ning G., Yang S., Yan Y., Cao Y., Wang Q. (2019). BMP Signaling Is Required for Nkx2.3-Positive Pharyngeal Pouch Progenitor Specification in Zebrafish. PLoS Genet..

[18] Crump J.G. (2004). An Essential Role for Fgfs in Endodermal Pouch Formation Influences Later Craniofacial Skeletal Patterning. Development.

[19] Yang S., Xu X., Yin Z., Liu Y., Wang H., Guo J., Wang F., Bao Y., Zhang T., Sun S. (2023). Nkx2.3 Is Responsible for Posterior Pharyngeal Cartilage Formation by Inhibiting Fgf Signaling. Heliyon.

[20] Klem J.R., Schwantes-An T.-H., Abreu M., Suttie M., Gray R., Vo H., Conley G., Foroud T.M., Wetherill L., CIFASD (2025). Mutations in the Bone Morphogenetic Protein Signaling Pathway Sensitize Zebrafish and Humans to Ethanol-Induced Jaw Malformations. Dis. Model. Mech..

[21] Flentke G.R., Klingler R.H., Tanguay R.L., Carvan M.J., Smith S.M. (2014). An Evolutionarily Conserved Mechanism of Calcium-Dependent Neurotoxicity in a Zebrafish Model of Fetal Alcohol Spectrum Disorders. Alcohol. Clin. Exp. Res..

[22] Stickney H.L., Imai Y., Draper B., Moens C., Talbot W.S. (2007). Zebrafish Bmp4 Functions during Late Gastrulation to Specify Ventroposterior Cell Fates. Dev. Biol..

[23] Chung W.-S., Stainier D.Y.R. (2008). Intra-Endodermal Interactions Are Required for Pancreatic β Cell Induction. Dev. Cell.

[24] Wada N., Javidan Y., Nelson S., Carney T.J., Kelsh R.N., Schilling T.F. (2005). Hedgehog Signaling Is Required for Cranial Neural Crest Morphogenesis and Chondrogenesis at the Midline in the Zebrafish Skull. Development.

[25] Bilotta J., Barnett J.A., Hancock L., Saszik S. (2004). Ethanol Exposure Alters Zebrafish Development: A Novel Model of Fetal Alcohol Syndrome. Neurotoxicol. Teratol..

[26] Everson J.L., Tseng Y., Eberhart J.K. (2022). High-throughput Detection of Craniofacial Defects in Fluorescent Zebrafish. Birth Defects Res..

[27] McCarthy N., Wetherill L., Lovely C.B., Swartz M.E., Foroud T.M., Eberhart J.K. (2013). Pdgfra Protects against Ethanol-Induced Craniofacial Defects in a Zebrafish Model of FASD. Development.

[28] Zhang C., Frazier J.M., Chen H., Liu Y., Lee J.-A., Cole G.J. (2014). Molecular and Morphological Changes in Zebrafish Following Transient Ethanol Exposure during Defined Developmental Stages. Neurotoxicol. Teratol..

[29] Lovely C.B., Nobles R.D., Eberhart J.K. (2014). Developmental Age Strengthens Barriers to Ethanol Accumulation in Zebrafish. Alcohol.

[30] Canfield D.V., Forster E.M., Cheong Z.-I., Cowan J.M. (2019). Breath/Blood Alcohol Concentration as an Indicator of Alcohol Use Problems. Aerosp. Med. Hum. Perform..

[31] Jones A.W. (2008). Ultra-Rapid Rate of Ethanol Elimination from Blood in Drunken Drivers with Extremely High Blood-Alcohol Concentrations. Int. J. Legal Med..

[32] Maier S.E. (2001). Drinking Patterns and Alcohol-Related Birth Defects. Alcohol Res. Health.

[33] Whaley C.C., Young M.M., Gaynor B.G. (2019). Very High Blood Alcohol Concentration and Fatal Hemorrhage in Acute Subdural Hematoma. World Neurosurg..

[34] Reimers M.J., Hahn M.E., Tanguay R.L. (2004). Two Zebrafish Alcohol Dehydrogenases Share Common Ancestry with Mammalian Class I, II, IV, and V Alcohol Dehydrogenase Genes but Have Distinct Functional Characteristics. J. Biol. Chem..

[35] Ibarra-García-Padilla R. (2021). Whole-Mount Immuno-Coupled Hybridization Chain Reaction (WICHCR): A Protocol for Dual Detection of mRNA and Protein Expression in Zebrafish Embryos and Early Larvae. STAR Protoc..

[36] Kuehn E., Clausen D.S., Null R.W., Metzger B.M., Willis A.D., Özpolat B.D. (2022). Segment Number Threshold Determines Juvenile Onset of Germline Cluster Expansion in *Platynereis dumerilii*. J. Exp. Zool. Part B.

[37] Schindelin J., Arganda-Carreras I., Frise E., Kaynig V., Longair M., Pietzsch T., Preibisch S., Rueden C., Saalfeld S., Schmid B. (2012). Fiji: An Open-Source Platform for Biological-Image Analysis. Nat. Methods.

[38] Bill B.R., Petzold A.M., Clark K.J., Schimmenti L.A., Ekker S.C. (2009). A Primer for Morpholino Use in Zebrafish. Zebrafish.

[39] Walker M., Kimmel C. (2007). A Two-Color Acid-Free Cartilage and Bone Stain for Zebrafish Larvae. Biotech. Histochem..

[40] David N.B., Saint-Etienne L., Tsang M., Schilling T.F., Rosa F.M. (2002). Requirement for Endoderm and FGF3 in Ventral Head Skeleton Formation. Development.

[41] Ruhin B., Creuzet S., Vincent C., Benouaiche L., Le Douarin N.M., Couly G. (2003). Patterning of the Hyoid Cartilage Depends upon Signals Arising from the Ventral Foregut Endoderm. Dev. Dyn..

[42] Johnson C.W., Hernandez-Lagunas L., Feng W., Melvin V.S., Williams T., Artinger K.B. (2011). Vgll2a Is Required for Neural Crest Cell Survival during Zebrafish Craniofacial Development. Dev. Biol..

[43] Smith S.M., Garic A., Berres M.E., Flentke G.R. (2014). Genomic Factors That Shape Craniofacial Outcome and Neural Crest Vulnerability in FASD. Front. Genet..

[44] Cartwright M.M., Smith S.M. (1995). Stage-Dependent Effects of Ethanol on Cranial Neural Crest Cell Development: Partial Basis for the Phenotypic Variations Observed in Fetal Alcohol Syndrome. Alcohol. Clin. Exp. Res..

[45] Rovasio R.A., Battiato N.L. (2002). Ethanol Induces Morphological and Dynamic Changes on In Vivo and In Vitro Neural Crest Cells. Alcohol. Clin. Exp. Res..

[46] McCarthy N., Eberhart J.K. (2014). Gene–Ethanol Interactions Underlying Fetal Alcohol Spectrum Disorders. Cell. Mol. Life Sci..

[47] Smith S.M., Garic A., Flentke G.R., Berres M.E. (2014). Neural Crest Development in Fetal Alcohol Syndrome: Neural Crest Development in Fas. Birth Defect. Res. C.

[48] Tsedensodnom O., Vacaru A.M., Howarth D.L., Yin C., Sadler K.C. (2013). Ethanol Metabolism and Oxidative Stress Are Required for Unfolded Protein Response Activation and Steatosis in Zebrafish with Alcoholic Liver Disease. Dis. Models Mech..

[49] Sidik A., Dixon G., Buckley D.M., Kirby H.G., Sun S., Eberhart J.K. (2021). Exposure to Ethanol Leads to Midfacial Hypoplasia in a Zebrafish Model of FASD via Indirect Interactions with the Shh Pathway. BMC Biol..

