# Supplementary material for: Ethanol Induces Craniofacial Defects in Bmp Mutants Independent of nkx2.3 by Elevating Cranial Neural Crest Cell Apoptosis
Source: Biomedicines. 2025 Mar 20;13(3):755. doi: 10.3390/biomedicines13030755 (PMC11940433; doi:10.3390/biomedicines13030755)
Supplement: Supplementary file 1 [file biomedicines-13-00755-s001.zip › biomedicines-3432114-supplementary.pdf]

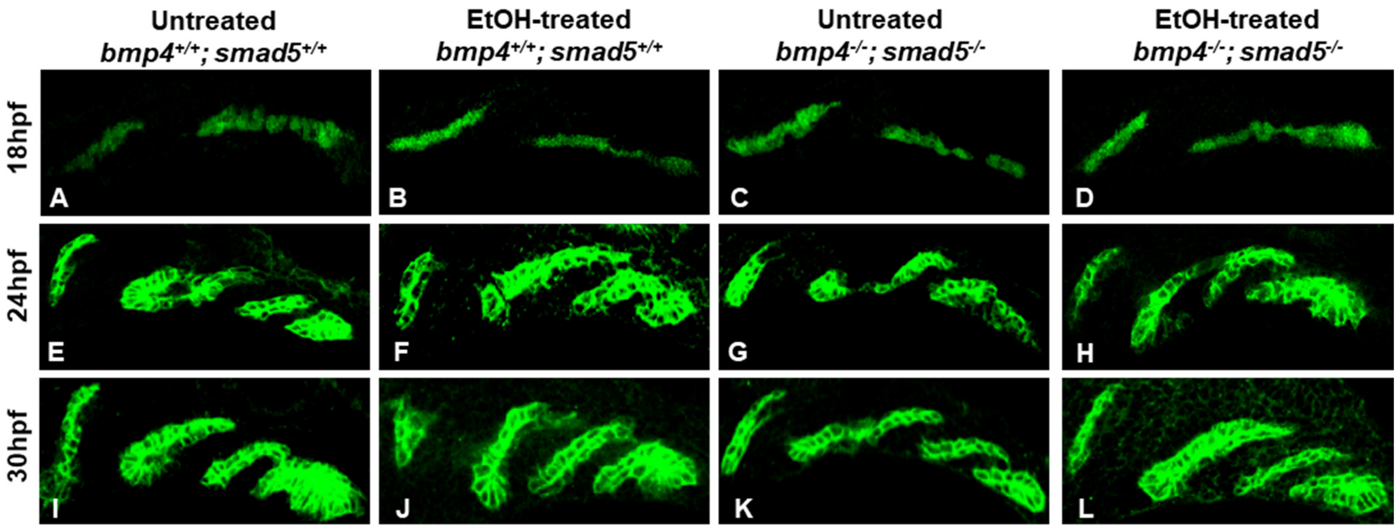

Supplement Figure S1: Confocal images of *sox17:EGFP*-labeled endodermal pouches in untreated and EtOH-treated wild type and *bmp4; smad5* double mutant embryos. Embryos were treated with ethanol from 10-18 hpf and confocal images of the endodermal pouches were taken at 18 hpf (A-D), 24 hpf (E-H), and 30 hpf (I-L). Pouches largely formed regardless of genotype or treatment, though their shape is no consistent.

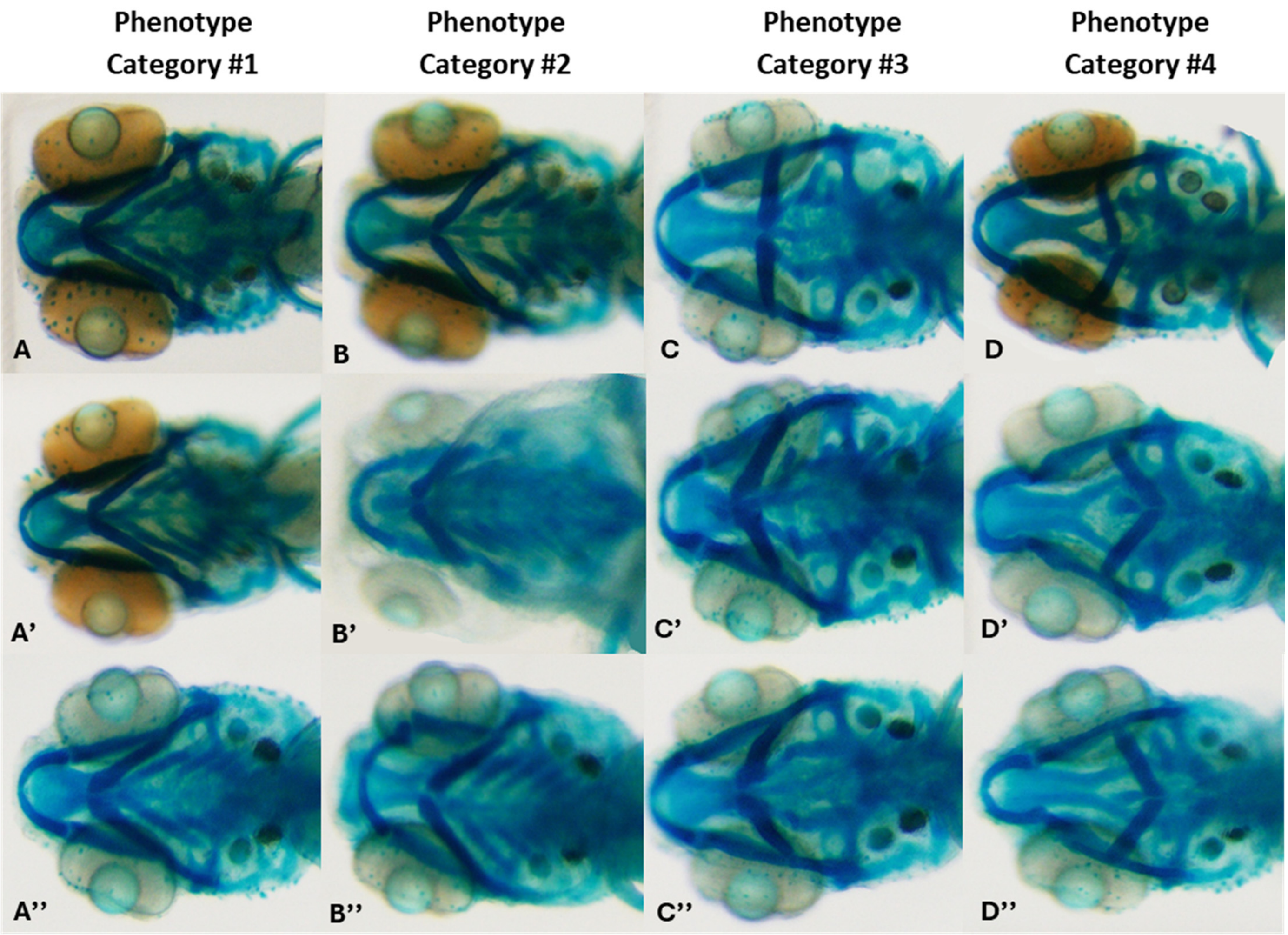

Supplement Figure S2: Representative images of phenotypic categories in nkx2.3-MO injected larvae. Four categories were used to assess the severity of facial defects. Category #1 = angle of ceratohyal (AOE) < 90º (A-A”). Category #2 = AOE from 90-135º (B-B”). Category #3 = 135 and 180° (C-C”). Category #4 = inverted ceratohyal (AOC > 180°, D-D”).

Supplemental Table S1. Ethanol-*bmp4-nkx2.3* three-way ANOVA statistics for Figure 4

ANOVA table	F (DFn, DFd)	P value
Treatment	F (1, 143) = 7.586	P=0.0066
Injection	F (1, 143) = 10.87	P=0.0012
Genotype	F (1, 143) = 26.25	P<0.0001
Treatment x Injection	F (1, 143) = 3.400	P=0.0673
Treatment x Genotype	F (1, 143) = 1.916	P=0.1684
Injection x Genotype	F (1, 143) = 5.160	P=0.0246
Treatment x Injection x Genotype	F (1, 143) = 5.033	P=0.0264

Control-MO	P Value	*nkx2.3*-MO	P Value
Untreated *bmp4*$^{+/+}$ vs. Untreated *bmp4*$^{-/-}$ - control-MO	0.9998	Untreated *bmp4*$^{+/+}$ vs. Untreated *bmp4*$^{-/-}$ - *nkx2.3*-MO	0.0002
Untreated *bmp4*$^{+/+}$ vs. Untreated *bmp4*$^{+/+}$ - *nx2.3*-MO	>0.9999	Untreated *bmp4*$^{+/+}$ vs. EtOH *bmp4*$^{+/+}$ - control-MO	>0.9999
Untreated *bmp4*$^{+/+}$ vs. Untreated *bmp4*$^{-/-}$ - *nkx2.3*-MO	<0.0001	Untreated *bmp4*$^{+/+}$ vs. EtOH *bmp4*$^{-/-}$ - control-MO	0.1126
Untreated *bmp4*$^{-/-}$ vs. Untreated *bmp4*$^{+/+}$ - *nkx2.3*-MO	0.9942	Untreated *bmp4*$^{+/+}$ vs. EtOH *bmp4*$^{+/+}$ - *nkx2.3*-MO	0.9728
Untreated *bmp4*$^{-/-}$ vs. Untreated *bmp4*$^{-/-}$ - *nkx2.3*-MO	<0.0001	Untreated *bmp4*$^{+/+}$ vs. EtOH *bmp4*$^{-/-}$ - *nkx2.3*-MO	0.0003
Untreated *bmp4*$^{-/-}$ vs. EtOH *bmp4*$^{+/+}$ - control-MO	0.9836	Untreated *bmp4*$^{-/-}$ vs. EtOH *bmp4*$^{+/+}$ - control-MO	0.0011
Untreated *bmp4*$^{-/-}$ vs. EtOH *bmp4*$^{-/-}$ - control-MO	0.0308	Untreated *bmp4*$^{-/-}$ vs. EtOH *bmp4*$^{-/-}$ - control-MO	0.9992
Untreated *bmp4*$^{-/-}$ vs. EtOH *bmp4*$^{+/+}$ - *nkx2.3*-MO	0.6775	Untreated *bmp4*$^{-/-}$ vs. EtOH *bmp4*$^{+/+}$ - *nkx2.3*-MO	0.0116
Untreated *bmp4*$^{-/-}$ vs. EtOH *bmp4*$^{-/-}$ - *nkx2.3*-MO	<0.0001	Untreated *bmp4*$^{-/-}$ vs. EtOH *bmp4*$^{-/-}$ - *nkx2.3*-MO	>0.9999
Untreated *bmp4*$^{+/+}$ vs. EtOH *bmp4*$^{+/+}$ - control-MO	0.9991		
Untreated *bmp4*$^{+/+}$ vs. EtOH *bmp4*$^{-/-}$ - control-MO	0.0443	EtOH *bmp4*$^{+/+}$ vs. EtOH *bmp4*$^{-/-}$ - *nkx2.3*-MO	0.0148
Untreated *bmp4*$^{+/+}$ vs. EtOH *bmp4*$^{+/+}$ - *nkx2.3*-MO	0.828		
Untreated *bmp4*$^{+/+}$ vs. EtOH *bmp4*$^{-/-}$ - *nkx2.3*-MO	<0.0001		
EtOH *bmp4*$^{+/+}$ vs. EtOH *bmp4*$^{-/-}$ - control-MO	0.1809		
EtOH *bmp4*$^{+/+}$ vs. EtOH *bmp4*$^{+/+}$ - *nkx2.3*-MO	0.9943		
EtOH *bmp4*$^{+/+}$ vs. EtOH *bmp4*$^{-/-}$ - *nkx2.3*-MO	0.0015		
EtOH *bmp4*$^{-/-}$ vs. EtOH *bmp4*$^{+/+}$ - *nkx2.3*-MO	0.4701		
EtOH *bmp4*$^{-/-}$ vs. EtOH *bmp4*$^{-/-}$ - *nkx2.3*-MO	0.9991		

Supplemental Table S1. Statistical analyses of Angle of in Figure 4. Angle of Ceratohyal was analyzed with a three-way ANOVA (type III). F-statistic and P-value for each analysis are shown. A Tukey's Multiple Comparisons Test for each comparison are shown.

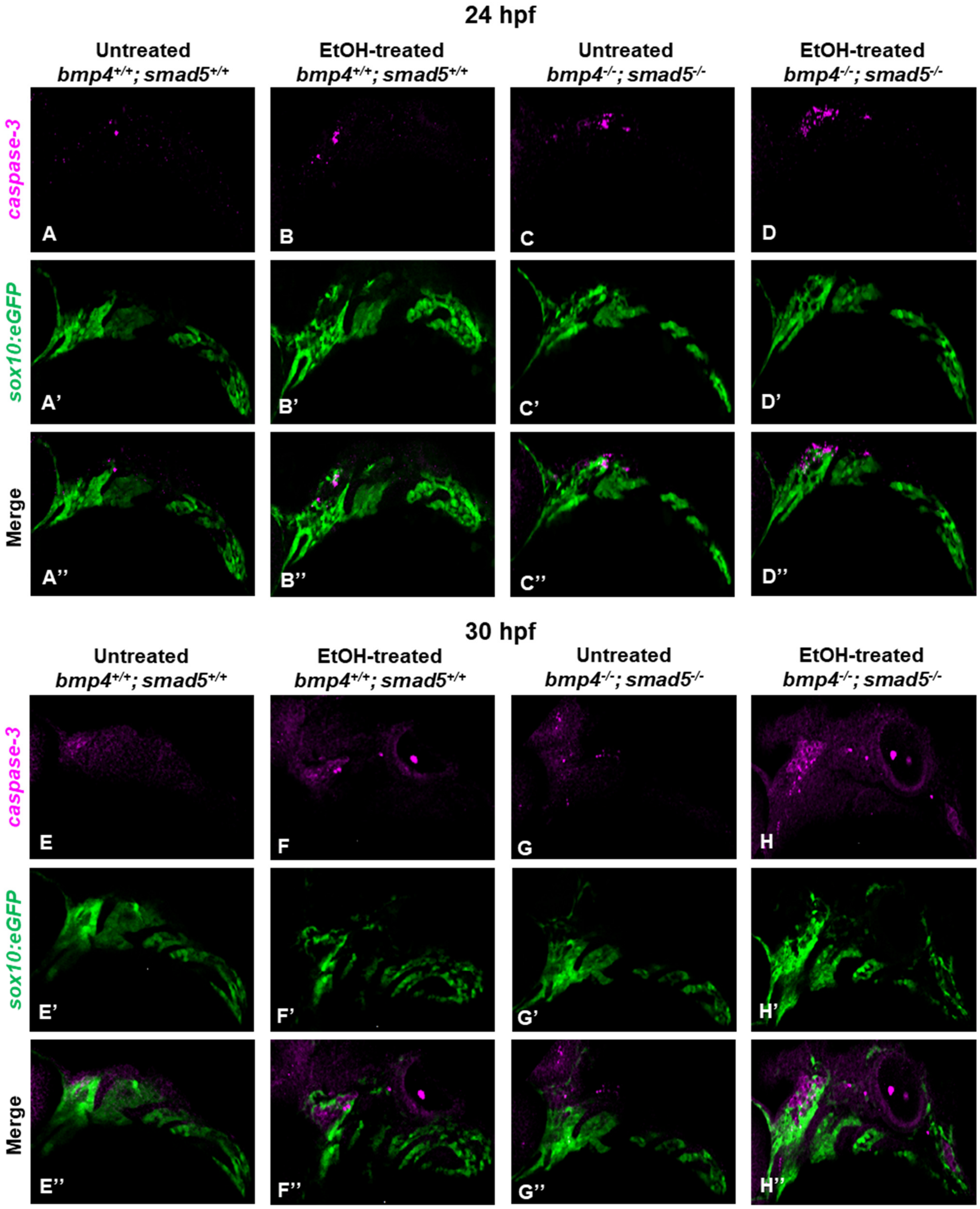

Supplement Figure S3: CNCC apoptosis persists to 30 hpf in ethanol-treated Bmp mutants. Confocal images of CNCCs (*sox10:eGFP*) and Cleaved Caspase-3 at 24 hpf (A-D") and 30 hpf (E-H"). Lateral views, anterior to the left in all images. We observed increased apoptosis in the CNCCs in ethanol-treated *bmp4; smad5* double mutant embryos compared to all other groups.
